# Morphogen interpretation: concentration, time, competence, and signaling dynamics

**DOI:** 10.1002/wdev.271

**Published:** 2017-03-20

**Authors:** Andreas Sagner, James Briscoe

**Affiliations:** ^1^ The Francis Crick Institute London UK

## Abstract

Tissue patterning during animal development is orchestrated by a handful of inductive signals. Most of these developmental cues act as morphogens, meaning they are locally produced secreted molecules that act at a distance to govern tissue patterning. The iterative use of the same signaling molecules in different developmental contexts demands that signal interpretation occurs in a highly context‐dependent manner. Hence the interpretation of signal depends on the specific competence of the receiving cells. Moreover, it has become clear that the differential interpretation of morphogens depends not only on the level of signaling but also the signaling dynamics, particularly the duration of signaling. In this review, we outline molecular mechanisms proposed in recent studies that explain how the response to morphogens is determined by differential competence, pathway intrinsic feedback, and the interpretation of signaling dynamics by gene regulatory networks. *WIREs Dev Biol* 2017, 6:e271. doi: 10.1002/wdev.271

For further resources related to this article, please visit the WIREs website.

## INTRODUCTION

Embryonic development is a progressive program in which a single totipotent cell, the fertilized egg, gives rise to hundreds of distinct differentiated cell types. For this to result in the successful completion of embryogenesis, and the well‐organized assembly of functioning organs, the appropriate cell types must be produced at the right time, at the right place and in the correct number. Fundamental therefore, is the elaboration of cell lineages in which multipotent progenitors are transformed to specific cell types in spatially stereotypic arrangements. Strikingly, a handful of inductive signals, iteratively used during development, coordinate this process. The repeated use of a limited set of signals means that the identity of a signal is not itself sufficient to confer specificity.

A key finding is that many inductive signals act as morphogens: locally produced, secreted signaling molecules that act over long distances and control growth and patterning throughout a region of tissue.^1^, ^2^ In these cases the response of receiving cells to the signal is dependent on their distance from the source of the signal, leading to the idea that morphogen concentration determines the downstream transcriptional program.^3^, ^4^, ^5^ This raises the question of how cells perceive and interpret different levels of the same signal. What mechanism transforms different levels of the signal into the distinct gene expression programs that determine different cell fates? This issue is further complicated by the observation that the timescales on which signals are transduced and gene expression patterns elaborated often do not match; tissue patterning usually occurs over several hours or days, whereas the signaling pathways usually function on the timescale of minutes reaching their maximum activity within a few hours, at most. Furthermore, tissue patterning typically occurs during phases of considerable tissue growth. Consequently, if the concentration of a morphogen is to be sufficient to impart positional information to the tissue, additional mechanisms are required to stably and accurately adjust morphogen activity to tissue growth over a prolonged time period. Thus, how precision in tissue patterning is reproducibly achieved during development remains an open question.

Recent findings from a number of systems have led to the realization that interpretation of developmental cues depends not only on the level but also the duration of signaling.^6^, ^7^, ^8^ In broad terms, the target genes induced or repressed in response to a signal depend on the current state of the receiving cells—its gene expression program—as well as the sensitivity of target genes to the signal. The changes in gene expression generated by the signal result in a new state of the cell and thereby alter the further possibilities available to the cell. Thus, the duration of a signal and the sequence in which a cell receives different signals influences its resulting fate. This highly context‐specific response highlights the importance of understanding how the temporal dynamics of a morphogen/signal, as well as its spatial behavior, pattern tissues.

In this review, we first discuss how differential competence underlies the specific response of cells to inductive signals. We then outline molecular mechanisms introducing dynamics into morphogen distribution and signaling and discuss how these dynamics affect signal interpretation. Finally, we highlight the role of transcriptional networks for integrating both levels and dynamics of morphogen signaling to control tissue patterning.

### Differential Competence Can Diversify the Response of Cells

#### *The Competence of Receiving Cells Determines How an Inductive Signal is Interpreted*

As outlined above, the iterative deployment of the same inductive signal in different developmental contexts suggests that interpretation of a signal is highly context‐dependent. This behavior was originally recognized in classic grafting experiments,^9^, ^10^, ^11^, ^12^ and led to the proposal that there are at least two components to each inductive event. The first element is the inducer (or evocator) defined as the signal emanating from a piece of tissue, such that when the tissue is transplanted to an ectopic position it elicits changes in the behavior of neighboring cells.^13^ Today, we understand that most of these inducers are secreted proteins and include morphogens belonging to the Wnt, Hedgehog (Hh), TGFβ, EGF, and FGF families. The second component is the competence of the receiving tissue to correctly interpret these signals to generate a specific outcome.^13^ This is exemplified by the observation that the inducer usually directs the induction of different cell types, depending on the spatiotemporal position in the embryo at which it is placed. A well‐established example of this is the transplantation of the floor plate (FP), the most ventral aspect of the neural tube, under the apical ectodermal ridge in chick wing buds. In the neural tube, the presence of a FP induces ventral neural cell types that include the generation of motor neurons.^14^ Yet when transplanted into the limb bud, FP tissue causes an axis duplication in the digit pattern.^12^ Thus in this case, FP transplants mirror the activity of the zone of polarizing activity (ZPA), a small part of mesodermal mesenchyme that instructs limb patterning along the anterior–posterior axis. This is due to both the FP and the ZPA patterning their respective tissues by secreting the same signaling molecule, Sonic Hedgehog (Shh).^15^, ^16^, ^17^, ^18^ Hence, the competence to respond to the secreted signal from the inducer (Shh from the FP or ZPA) to specifically produce an outcome (motor neuron generation vs digit patterning) resides in the gene expression profile of receiving cells.

#### *Molecular Mechanisms for Mediating Competence*

**Figure 1:**
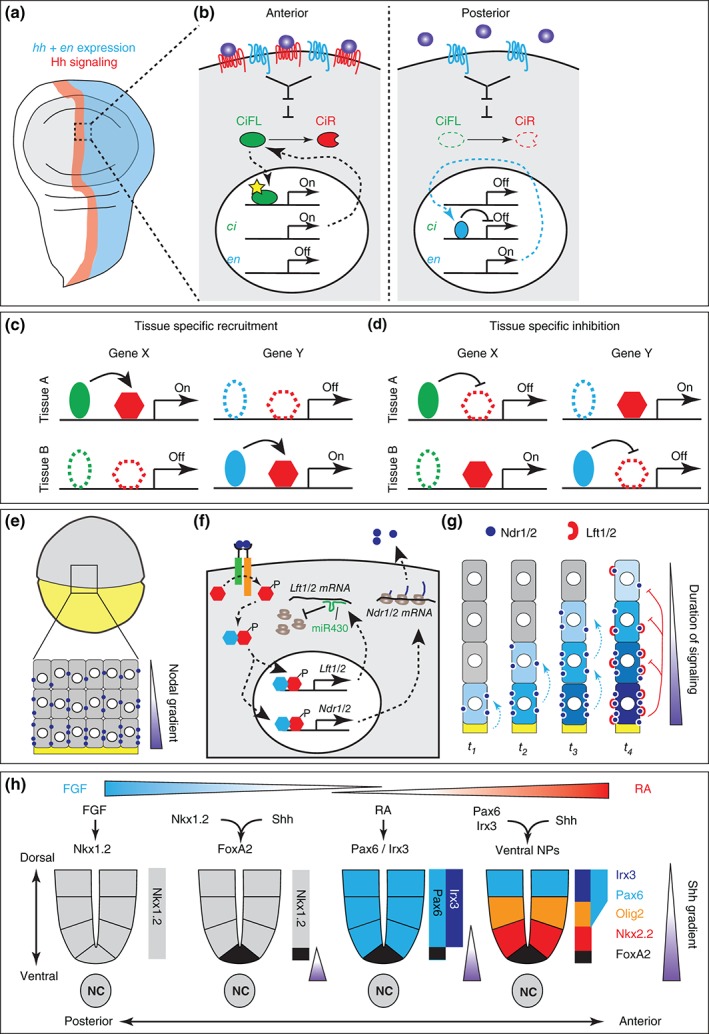
Differential competence for morphogen interpretation resides in the gene expression profile of receiving cells. (a) Scheme of a Drosophila wing disc displaying the subdivision into anterior and posterior compartments. The posterior compartment (blue) is characterized by expression of engrailed (en) and hedgehog (hh). Secreted Hh protein diffuses anteriorly, where it signals in a small stripe of cells (red). (b) En in the posterior compartment represses expression of ci, the transcriptional mediator of the Hh pathway. In contrast, cells in the anterior compartment express ci and can therefore activate Hh signaling. Hh binds and thereby inhibits the activity of Ptc (red), which relieves inhibition of Smo (blue). Smo activity blocks the proteasomal degradation of full‐length ci (CiFL) to its repressor form (CiR) and instead activates ci to promote the expression of Hh target genes. (c) Tissue specific activation of target genes by recruitment of transcriptional effector proteins (red hexagons, e.g. Gli) to specific binding sites in the genome by tissue specific cofactors (green and blue ellipses, e.g. SoxB family members in the neural tube). (d) Tissue specific inhibition of target genes by TFs that block the access of transcriptional effectors to specific binding sites (e.g. REST in non‐neuronal cells). Absence of these TFs leads to target gene activation. (e) Scheme of a zebrafish embryo at mid‐epiboly. (f) Nodal signaling in cells activates expression of Nodal ligands Ndr1/2 and the pathway inhibitors Lft1/2. The temporal competence window for Nodal signaling arises by miR430 delaying the translation of the pathway inhibitors Lft1/2. (g) Nodal signaling from the yolk syncytial layer (yellow) initially induces Ndr1/2 expression in cells directly at the margin (t_1_). Nodal signaling then spreads to its immediate neighbors, where it induces expression of Nodal ligands (t_2_, t_3_). This sequential induction of Nodal ligands and signaling results in a temporal gradient of Nodal signaling in marginal cells. The window for further spreading of Nodal signaling is terminated when Lft1/2 translation overcomes inhibition by miR430 (t_4_). (h) The differential competence for FP induction in response to Shh is mediated by opposing FGF and RA gradients along the anterior–posterior axis of the embryo. High levels of FGF signaling in the tail bud promote expression of Nkx1.2 (left). Combined activity of Nkx1.2 and Shh signaling in ventral parts of the neural tube induce the expression of FoxA2, which specifies FP cells (middle left). As cells are displaced anteriorly during development, they start to express Pax6 and Irx3 in response to RA, which then represses Nkx1.2 (middle right). Shh signaling in Pax6/Irx3 expressing cells induces expression of ventral neural progenitor markers Olig2 and Nkx2.2 (right).

How is competence mediated at the molecular level? In its simplest instance, competence is imposed by the expression of all the necessary components for receiving a signal. Loss of competence results if parts of a signal transduction cascade are missing or blocked. This is for example the case for cells in the Drosophila wing disc. Cells in the anterior compartment of the wing disc express *cubitus interruptus* (*ci*), the transcriptional effector of the Hh pathway,^19^, ^20^, ^21^ and respond to Hh (Figure 1(a) and (b)). By contrast, cells in the posterior compartment lack expression of *ci* 22, 23 and therefore are not able to respond to Hh, despite its presence in high concentrations (Figure 1(b)). This difference in competence to respond to Hh signaling is critical for the initial subdivision of Drosophila segments into anterior and posterior compartments and their subsequent maintenance throughout development.

This mechanism, however, only allows the discrimination between ON and OFF states of the signaling pathway and does not explain the induction of a distinct set of target genes in different tissues in response to the same signaling molecule. This phenomenon, in principle, could be explained by signals acting through different context‐dependent receptors or transcriptional effector proteins. Such a mode of action has been for example demonstrated for bone morphogenetic protein (BMP) signaling in the dorsal neural tube.^24^, ^25^ Here, BMP signaling through BMP receptor 1a (BMPR1a) induces dorsal identity and proliferation of neural precursor cells. Furthermore, it also leads to expression of a second BMP receptor, BMPR1b. Activation of BMPR1b in turn drives cells out of the cell cycle through induction of p21^CIP1^ and induces neuronal differentiation.^25^ In most cases, however, signaling pathways and transcriptional mediators are conserved between tissues and competence states. Thus, in these cases the solution to the problem must lie in the differential recruitment of transcriptional effector proteins to varying sites in the genome, depending on the tissue context (Figure 1(c)). Such behavior has been demonstrated for the activation of neural specific Shh and BMP target genes, which depends on the presence of neural specific transcription factors (TFs) of the SoxB family.^26^, ^27^, ^28^ Consistent with this, many neural specific Shh target genes seem to be controlled by cis‐regulatory motifs (CRMs) in which Sox and Gli binding sites are in close proximity.^26^, ^27^, ^28^, ^29^, ^30^ Furthermore, ectopic expression of SoxB family members in the limb bud is sufficient to induce neural specific Shh target genes in this tissue.^27^ These results indicate that neural competency in a tissue in response to Shh is dependent on the availability of SoxB family members.

In addition to the role of sequence specific TFs, epigenetic changes, such as alterations in chromatin modifications, can modify the accessibility or operability of genomic regulatory elements. One of the best‐described mediators for such epigenetic changes during development is the TF REST (RE1 silencing transcription factor; also called NRSF), which orchestrates the repression of neuronal genes in non‐neuronal tissues.^31^, ^32^, ^33^, ^34^ This function is of critical importance for proper vertebrate development, as REST mutant mice die at early embryonic stage and display neuronal gene expression in non‐neural tissues.^35^ Upon neuronal differentiation, REST is downregulated and selectively degraded.^36^ The protein consists of two independent repressor domains, one at the N‐ and C‐terminus, respectively, and a central DNA binding domain harboring eight zinc‐finger motifs that recruit REST to neuronal genes through binding to a 21 bp conserved DNA motif called RE1 (repressor element 1; also called NRSE).^32^, ^33^, ^37^ The N‐terminal repressor domain interacts with mSin3 and recruits histonedeacetylases (HDACs) to mediate short‐term inactivation of neuronal genes.^38^ In contrast, the C‐terminal repressor domain interacts with the corepressor CoREST which recruits besides HDACs also histone H3—lysine 9 (H3K9) methyltransferase G9a and H3K4 demethylase LSD1.^31^, ^39^, ^40^, ^41^, ^42^, ^43^ Furthermore, the CoREST complex also recruits MeCP2 (methyl‐CpG binding protein 2) and DNMT1 (DNA methyltransferase 1) leading to DNA methylation.^36^, ^41^ The combined enzymatic activities of these proteins generate binding sites for HP1 (heterochromatin protein 1) and result in chromatin condensation and consequently inactivation.^31^, ^41^ REST‐mediated gene silencing does not seem to be limited to genes in the vicinity of RE1 sites. Rather these sites act as nucleation centers for the downregulation of entire chromosomal intervals harboring multiple neuronal genes.^41^ In summary, tissue specific gene expression in this case is achieved through a specific epigenetic repressor complex present in all tissues where the corresponding target genes are not supposed to be expressed (Figure 1(d)).

In many cases such epigenetic mechanisms also confer memory to cells and are critically required for the maintenance of cell states.^44^, ^45^ Elegant *in vitro* work revealed that the timescales of epigenetic memory depend on the type of modification and the duration of recruitment of modifying enzymes to genomic loci and can easily exceed several weeks.^46^ Therefore, such epigenetic modifications can confer memory over much longer timescales than gene regulatory interactions through sequence‐specific TFs. Well known examples are the previously discussed REST complex, which mediates long‐term silencing of at least some neuronal genes in non‐neuronal tissues,^34^, ^36^ or the stable silencing of core pluripotency genes following embryonic stem cell differentiation (reviewed in Ref 44). Taken together, epigenetic modifications can mediate competence over long timescales and thereby provide a mechanism by which competence does not only depend on the current transcriptional profile of a cell but its entire history.

#### *Temporally Limited Competence Can Arise From Negative Feedback or Changes in the Signaling State of a Cell*

In most cases, competence to a signal defines specific developmental stages. Such ‘competency windows’ can arise in response to pathway‐intrinsic feedback, resulting in self‐limiting competency. An example of how this provides a temporal window for competence is the delayed expression of a pathway specific inhibitor. This appears to be the case for mesendoderm specification in response to Nodal signaling in the zebrafish embryo^8^ (Figure 1(e)). Here, the Nodal ligands Ndr1 and Ndr2 induce expression of their antagonists Lefty 1 (Lft1) and Lefty2 (Lft2), which blocks further signaling (Figure 1(f) and (g)). Importantly, translation of Lft1 and Lft2 are delayed by activity of a micro‐RNA, miR‐430 (Figure 1(f)). Inhibition of miR430 activity results in premature Lft1/2 expression and consequently reduced levels of Nodal signaling. Thus, the miR430‐mediated delay in the accumulation of Lft1 and Lft2 thereby generates a temporal window for Nodal ligands to induce target genes (Figure 1(g)). This mechanism has been suggested to dictate the overall size of the Nodal signaling domain. In this view, Nodal ligands only act on adjacent cells where they induce their own expression, this propagation is terminated when signaling is inhibited by Lft1/2^8^ (Figure 1(g)).

Windows of competence can also arise from the temporal sequence of different signals or by continuous exposure to the same signal. For example, cells in the neural tube can differentiate into FP in response to Shh or roof plate (RP) in response to BMP signaling.^47^ The competence of Shh and BMP to induce these cell types is controlled by receiving cells responding to opposing gradients of FGF and retinoic acid (RA). In the posterior part of the embryo, high levels of FGF signaling induce expression of the TF Nkx1.2^48^ and in this state cells respond to Shh and BMP by inducing FP and RP, respectively (Figure 1(h)).^47^ As axis elongation displaces cells anteriorly, FGF signaling and consequently expression levels of Nkx1.2 decline. This allows cells to induce Pax6 and Irx3 in response to RA,^49^, ^50^ thereby terminating the competence of cells to induce FP or RP in response to Shh or BMP, respectively (Figure 1(f)). Instead these signals induce the progenitors of distinct neuronal subtypes.^47^ Importantly, this change in competence does not impede the ability of cells to respond to Shh or BMP, but changes how these signals are interpreted by the underlying gene regulatory network (GRN). Thus, in this example, there is a temporal window of competence for cells to form FP and RP within the neural plate that is defined by the range of FGF and RA gradients acting orthogonally to the inducing signals BMP and Shh (Figure 1(h)). This example illustrates a more general concept of development, where a signal or a change in signals (reduction of FGF, exposure to RA) alters the gene expression profile of a cell (from Nkx1.2 to Pax6/Irx3), thereby changing the competence to produce a specific outcome (induction of FP and RP). Crucially such changes in competence are not necessarily accompanied by a change in the signaling state of a cell.

Equally, longer exposures to signals can lead to cascades of gene activation, where the competence to induce target genes of the next step, depends on target genes activated in the previous—a mechanism dubbed ‘sequential cell context.’^51^ Thus, the duration of exposure to the signal gradually changes the competence of cells over time. We outline below how this behavior underpins the interpretation of signaling dynamics, especially duration, by GRNs.

### How Cells Interpret Signaling Dynamics—A Mechanism for Diversity

In the previous section, we outlined how differences in cell competence can produce different cell fates in response to the same inducing signals. In the following sections, we will focus on how signaling dynamics affect cell fate decisions, and thus allow further diversification of the repertoire of cell types that can form in a given tissue.

Dynamics in morphogen signaling can arise in three main ways: (1) the inductive signal itself can have a dynamic pattern of production or movement through the tissue; (2) positive and negative feedback in the signaling pathway can modulate the response of cells to the ligand; or (3) the underlying GRN can interpret and influence the dynamics of the signal.

In the conventional view of morphogen activity all information for differentially inducing target genes is provided by the concentration of the ligand producing different levels of signal transduction. In addition to the level, however, dynamical properties of a signal can be used to carry information—the fold change in signal level, the duration, the amount of signal accumulated over time (integral) and the rate of change of signaling (the speed at which the level increases or decreases). Additionally, if a signal oscillates, the number and frequency of the oscillations can also communicate information. Obviously, these combinations are not mutually exclusive. For example, a signal might need to exceed a certain threshold within a certain time period to induce a specific response, and exceeding this threshold at later stages does not result in the same response.

### Dynamics in Morphogen Distribution

#### *Morphogen Gradients are Both Spatial and Temporal*

Inductive signals are not only gradients in space, but also in time. How a morphogen is produced and disseminated can lead to dynamics in signaling. The rate of production, rate of clearance in receiving cells, and the speed of movement/diffusion determine the distribution of a morphogen in a tissue.^2^, ^4^, ^52^ Changes in any of these properties during tissue patterning can contribute to levels of the signal changing in the tissue. Increases or decreases in the rate at which a morphogen is produced from its source results in changes in the amplitude of the signal. If the morphogen spreads rapidly and is cleared rapidly from the tissue then changes in production level rapidly yield steady state changes in the gradient throughout the tissue. However, if the spread or clearance of the ligand happens over relatively long timescales it will take a period of time for the gradient to reach its new steady state. During this pre‐steady state period the levels of morphogen will change at different rates at different positions within the tissue.^53^ For example, shortly after morphogen production has been initiated, cells closest to the source receive ligand first and the levels of ligand at these positions increase over time. Consequently, target genes requiring lower thresholds for activation usually appear first close to the morphogen source and the tissue experiences increasing concentrations of the morphogen over time. This dynamic pattern of target gene induction has been observed in several tissues, for example for Nodal signaling in the early zebrafish.^54^

The extent to which morphogen gradients are pre‐steady directly depends on the persistence time of the morphogen in the tissue and the speed with which patterning is read out from the gradient. The longer the persistence time and the faster the readout speed by the patterning system, the more likely it is that pre‐steady state dynamics of the morphogen govern patterning. Pre‐steady state decoding has been proposed for the Bicoid gradient in the Drosophila embryo.^53^, ^55^ Examples of adiabatic gradients—in which changes in amplitude do not appear to result in protracted pre‐steady intervals—have been described for Shh in the neural tube^56^, ^57^ and for Dpp in the Drosophila wing disc.^58^, ^59^

#### *Morphogen Dynamics Emerging From Spatially Dynamic Expression*

**Figure 2:**
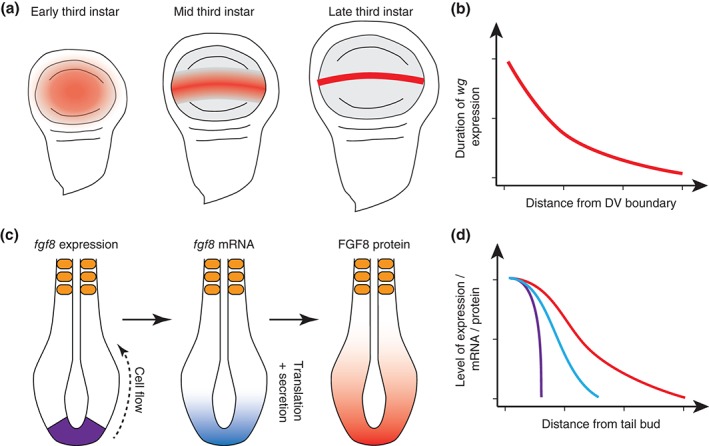
Spatially dynamic morphogen expression. (a) Wg expression (red) initially broadly expressed in the Drosophila wing pouch recedes to the DV boundary during development. (b) A temporal gradient of Wg expression in the wing disc underlies the complete rescue of wing patterning by a nondiffusible Wg version. (c) Formation of a fgf8 mRNA gradient during axis elongation in vertebrate embryos. Expression of the fgf8 is restricted to posterior regions of the tail bud (violet). As cells get displaced anteriorly during axis elongation they carry fgf8 mRNA with them, leading to a fgf8 mRNA gradient (cyan). This gradient is continuously translated into a gradient of FGF8 protein (red). (d) Levels of fgf8 gene expression (violet), fgf8 mRNA (cyan), and FGF8 protein at different distances from the tail bud.

Another way in which temporal gradients can be generated is by spatial changes in the expression pattern of the morphogen. Such expression dynamics might in some cases account for the fact that movement of the morphogen appears dispensable for patterning. One such case is Wingless (Wg) in the Drosophila wing disc.^6^ In late stage wing discs, Wg is expressed at the dorsal–ventral (DV) compartment boundary from where it is secreted and forms a gradient along the dorsoventral axis of the wing. This suggested that an extracellular gradient of Wg controls wing disc development. Surprisingly, however, a membrane‐tethered version of Wg is sufficient for patterning and growth of the wing disc. How can this result be reconciled with previous studies^60^, ^61^ that demonstrated a requirement for graded activity of Wg for patterning and growth of the wing disc? Strikingly, at early larval stages, *wg* is transcribed throughout the entire prospective wing field and its expression only later recedes to the DV boundary (Figure 2(a)). These changes in Wg expression generate a temporal gradient throughout the wing pouch—compared to cells at the DV boundary, cells at the lateral edge of the pouch only express and receive Wg briefly at early developmental time points (Figure 2(b)). These differences in dynamics presumably suffice to pattern the wing along the DV axis. Similarly, Nodal signaling in the early zebrafish has been recently proposed to form a temporal rather than a spatial gradient.^8^ Similar to Wg, van Boxtel et al.^8^ suggest that the inductive range of Nodal is limited to one row of cells adjacent to its expression domain (Figure 1(g)) (although Nodal ligands can diffuse and induce target genes at a distance from the source when ectopically expressed^62^). These examples epitomize how the formation of temporal, instead of spatial gradients, may in part underlie the action of many morphogens.

Spatial gradients in morphogen production can also emerge from the displacement of cells during development, especially, in growing tissues.^63^, ^64^, ^65^ This is for example the case for FGF8 during vertebrate axis elongation.^63^ Here, transcription of *fgf8* mRNA is restricted to the posterior tip of the embryo (Figure 2(c) and (d)). As progressively new tissue is formed from this growth zone, cells move out of the zone of *fgf8* transcription, but carry *fgf8* mRNA with them (Figure 2(c) and (d)). Due to mRNA degradation, the level of *fgf8* mRNA per cell is proportional to the time a cell spend outside the zone of *fgf8* induction and therefore to the distance of the cell to the region *fgf8* induction. Thus, in this case the anterior displacement of cells due to axis elongation directly results in the formation of a *fgf8* mRNA gradient, which is continuously translated into a gradient of FGF8 protein (Figure 2(c) and (d)). As the FGF8 gradient directly controls differentiation,^50^, ^66^, ^67^ this mechanism allows efficient coupling between the speed of posterior axis elongation and differentiation in response to FGF signaling. A similar mechanism has been proposed for Wnt3 in the gut intestinal stem cell niche.^65^ Here Wnt3 is expressed in specialized cells called Paneth cells, which provide a pool of Wnt3 at the plasma membrane to stem cells in their immediate neighborhood. Similar to Wg in the wing imaginal disc, Wnt3 dissemination in this system is not by diffusion; instead Wnt3 spread depends on cell divisions that dilute the pool of surface‐bound Wnt3 proteins.^65^

### Generation of Dynamics in the Signal Transduction Pathway

#### *Linear Signaling Pathways*

The dynamics of signaling in cells receiving a morphogen do not only arise in response to changes in the concentration of external ligand. In many cases the mechanism of signal transduction can introduce dynamics that decouple the direct connection between ligand concentration and the output of the pathway.

In the simplest instances, a signaling pathway transduces a signal from the plasma membrane to the nucleus without any nonlinear amplification or feedback. Examples of this type of signaling have been reported. For example, a threefold difference in the number of activated Activin receptors appears to be translated in a threefold difference in the number of nuclear Smad2 during the differential activation of Brachyury (Bra) and Goosecoid (Gsc) in *Xenopus* blastula cells.^68^, ^69^ Similarly, the gradient of Dpp in the Drosophila wing disc seems to be converted into a similarly shaped gradient of phosphorylated Mothers against Dpp (pMad) in the nucleus.^70^ Theoretically, in these cases the output of the pathway is determined by the concentration of ligand, the receptor, the binding kinetics and the capacity of the downstream signaling cascade to transduce the signal. In these terms the activity of the downstream effector can be directly proportional to the concentration of the upstream ligand. However, such linear systems have potential disadvantages. All components of the signal transduction cascade have to be present at levels sufficient for maximal pathway activation even in the absence of signal. Moreover, the system is susceptible to noise at all levels (noise in ligand distribution in the tissue, ligand–receptor interactions, and transduction through the intracellular signaling pathway).

#### *Emergence of Pathway Intrinsic Dynamics from the Molecular Mechanisms of Signal Transduction*

The mechanisms employed within the signaling pathway can introduce dynamics. For example, both the Wnt and Hh signaling pathways, which are used in several developing tissues, exploit the irreversible degradation of the transcriptional effectors of the pathway, either completely degrading them or proteolyzing them into repressors in the absence of signal.^71^ Thus, on exposure to the ligand, the level of activation of the pathways depends not only on the amount of ligand, but also on the rates of *de novo* production of the effector proteins and the degradation of the previously formed repressors. This effectively slows the transduction of the signal and imparts some resistance to rapid fluctuations in the level of ligand. Similarly, ligands can stay bound to and activate receptors over a longer timescale, thereby allowing cells to ‘remember’ a signal over a certain timescale. Such a mechanism has been proposed for Activin signaling in *Xenopus* blastula cells. Here cells can remember hours later Activin exposures that were as short as 10 min.^72^, ^73^ At the molecular level this behavior may be caused by Activin staying bound to its receptor on the timescale of several hours^69^ and endocytosis of the ligand–receptor complex.^74^ Similarly, in Drosophila the BMP homologue Dpp localizes together with its receptor to specialized endosomes labeled by the endosomal protein Sara, where it recruits R‐Smads to the type 1 receptor Thickveins for phosphorylation.^75^ During cell division, Sara endosomes associate with the spindle and thereby allow for the equal inheritance of signaling components between daughter cells.^75^ Endocytosis has also recently been proposed to underlie continuing Nodal signaling in zebrafish blastula cells after treating cells with the Nodal inhibitor Lefty.^8^ In these examples the molecular mechanism of signaling effectively acts as a high frequency filter that allows cells to ignore changes in signaling occurring at short timescales.

Analyses of how much information can be communicated at any one moment by signaling pathways emphasize the limited capacity to control multiple decisions.^76^ This raises the question of how the cell interprets a morphogen signal and how signaling dynamics are used in support of this outcome. Specifically, it is important to understand the mechanisms that convert a temporally changing morphogen gradient into discrete boundaries of gene expression that are necessary to generate distinct cell types at appropriate locations.

#### *Zero‐Order Ultrasensitivity and Positive Feedback Can Generate Switch‐like Behavior From a Graded Input*

Several mechanisms have been described that introduce specific nonlinearities into a signal. Ultrasensitivity and positive feedback result in the conversion of a continuous graded signal into a step‐like signal, whereas negative feedback allows cells to adapt to a signal. Besides affecting the output of a signaling pathway, these mechanisms have been shown to control the shape of morphogen gradients and buffer them against fluctuations. Consequently, disruption of these feedback mechanisms usually results in abnormal patterning. Thus, these feedback mechanisms are a vital part of their patterning systems and their function is indispensible for inducing expression domains at the right place, size, and time.

Ultrasensitivity can arise when two enzymes with opposing activities (e.g. a kinase/phosphatase pair) are saturated by their substrates.^77^ The consequence is that the enzymes operate with so‐called zero‐order kinetics, in which the rate of the reactions is nearly independent of substrate concentration. If the enzymes target a protein modification, then at steady state the target protein pool will be in either one state or the other, depending on the difference in the rates of the forward and backward reactions.^77^ In this situation small changes in the enzymes activities will completely change the state of the substrate. Thus, if the activity or concentration of one of the modifying enzymes is controlled by a graded input, these systems can generate step‐like changes in the behavior of the substrate.^53^, ^78^ Such a mechanism has been described for patterning of the Drosophila ventral epidermis.^79^ Here graded input of the EGF ligand Spitz generates a gradient of MAP Kinase activity, which promotes degradation of the TF Yan in response to phosphorylation. Furthermore, using a combination of experimental approaches and theory, it has been demonstrated that the Yan phosphorylation cycle generates a zero‐order ultrasensitvity loop with the capacity to form sharp boundaries of Yan expression in the tissue.^79^

Another way in which graded signals can be converted into step‐like cellular responses is via positive feedback of the signaling pathway, where high levels of signaling enhance further signaling, for example by promoting ligand–receptor interactions, and low levels of signaling result in a downregulation of signaling capacity. Such a mechanism has been proposed to explain signaling activities of the BMP ligands Dpp and Screw during dorsoventral patterning in Drosophila.^80^ In both cases pathway‐intrinsic dynamics provide a means for converting initially graded input into an ON/OFF switch that could be used to control the position at which a target gene is activated and thereby generate a defined border within the forming tissue. However, a limitation of these mechanisms is that they can only result in a single ON/OFF step.

### Negative Feedback Causes Pathway Adaptation

#### *Advantages of Negative Feedback for Signal Interpretation*

One of the most commonly used mechanisms for temporal signal integration is negative feedback, in which a signaling pathway actively promotes expression of an inhibitor of the signal transduction cascade. In many cases these inhibitors are considered to be canonical target genes of the respective signaling pathways, and their expression levels are proxies for levels of pathway activity (e.g. Ptch1 for Hh signaling, Axin2 and Notum for Wnt signaling, Sprouty protein members for FGF signaling, Argos induction in response to EGF signaling, and Hes1/5 for Notch signaling). Such negative feedback systems confer several properties, namely they can increase the dynamic range of ligand a cell can sense, lead to pathway adaptation and provide a means for cells to measure duration of signaling. Another form of negative feedback is ligand‐mediated receptor degradation, which has been described for EGF and TGFβ signaling.^81^, ^82^

A negative feedback inhibitor that directly interacts with the ligand or the receptor can allow the cell to increase the range of ligand concentrations it can measure with the same number of receptors, as high levels of ligand promote strong production of inhibitors, which will sequester the number of available ligands.^2^, ^83^ Consequently, the concentration of ligand a cell is exposed to must continuously increase to maintain stable levels of signaling in the cell, and failure to do so will cause the signaling pathway to adapt to the input signal, that is the activity of the signaling pathway will decrease despite the presence of ligand. Similarly, if the inhibitor acts on the downstream signaling cascade, its induction can be used to inactivate the pathway after a certain amount of time in response to the signal. Such a mechanism is useful for discerning the duration of ligand exposure or the rate at which ligand concentration increases. These mechanisms allow cells to convert the concentration of ligand into duration of signaling and to measure signaling dynamics. We will outline these ideas using two examples from the recent literature: adaptation to Shh signaling in the ventral neural tube, which allows cells to effectively measure the integral of signaling activity,^7^, ^57^, ^84^ and adaptation in the response to TGFβ signaling, which provides a means to measure the rate of increase in ligand concentration.^85^, ^86^

#### *Adaptation of the Shh Pathway Allows Cells to Integrate Levels and Duration of Pathway Activity*

**Figure 3:**
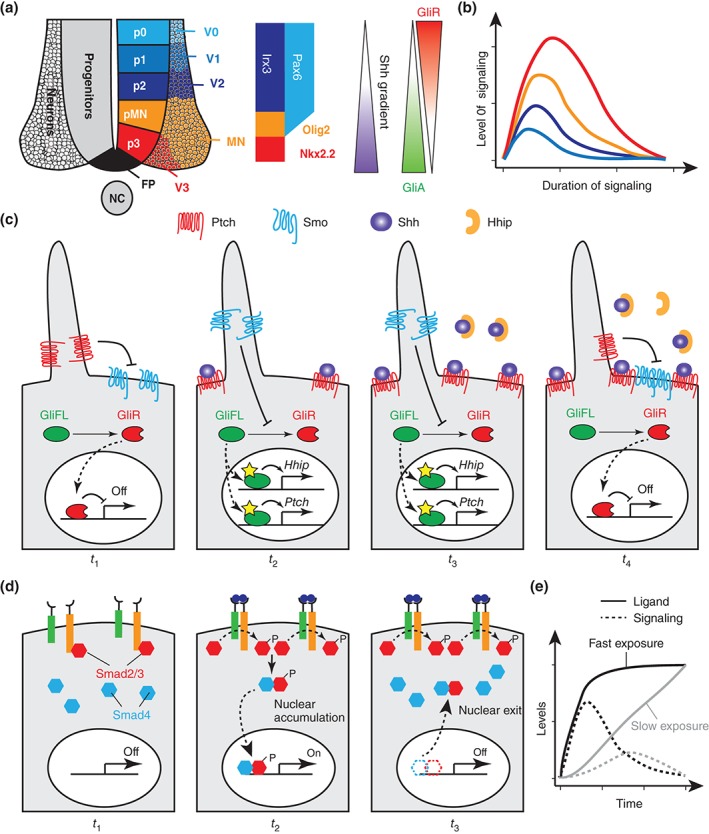
Pathway adaptation as means to measure signaling duration and dynamics. (a) The ventral neural tube is subdivided into different progenitor domains (p0–p3 and pMN) which form distinct classes of interneurons (V0–V3) and motor neurons. Subdivision of the three most ventral progenitor domains (p2, pMN, p3) is controlled by the differential expression of Irx3 (p2 only), Pax6 (p2, low in pMN), Olig2 (pMN), and Nkx2.2 (p3). This pattern is established in response to the graded activity of Shh, which generates two opposing gradients of activated Gli (GliA) and Gli repressor (GliR). (b) Due to pathway intrinsic negative feedback, different levels of Shh signaling are converted into distinct durations of Shh pathway activity. (c) Pathway intrinsic negative feedback leads to Shh pathway adaptation. In the absence of ligand (t_1_), Ptch1 receptors block the entrance of Smo to the primary cilium. This leads to proteasomal degradation of full‐length Gli (GliFL) into its repressor version (GliR). Upon exposure to Hh ligands (t_2_), Hh binds to Ptch1 and inhibits its activity, so Smo can enter into the cilium. Smo activity blocks Gli processing leading to stabilization and activation of GliFL, which activates target genes in the nucleus. Among the target genes of Gli are Hhip1 and Ptch1, which encode negative regulators of Hh pathway activity. Consequently, levels of Hh ligands need to rise continuously to inactivate increasing concentrations of Hhip1 and Ptch1 receptor (t_3_). Failure to do so, results in sequestration of Hh ligands and inactivation of the pathway (t_4_). (d) Pathway adaptation can be used to measure the speed of ligand exposure in the TGFβ pathway. In the absence of ligand, TGF receptors do not dimerize and Smad2/3 stay bound to the receptors (t_1_). Upon ligand binding, receptors dimerize, and phosphorylate Smad2/3, which allows dissociation of phosphorylated Smad2/3 from the receptor, interaction with Smad4 in the cytoplasm, nuclear accumulation, and activation of target genes (t_2_). Despite continuous exposure to the ligand, Smads are transported from the nucleus after a certain amount of time, leading to pathway adaptation (t_3_). (e) Pathway adaptation can be used to measure the speed of ligand exposure. Fast exposure of ligand (solid black line) leads to high levels of signaling (dashed black line) before the pathway adapts. In contrast, the response to slower ligand exposure (gray lines) is dampened by pathway adaptation.

In the vertebrate spinal cord neuronal progenitor domains are specified along the DV axis by the distinct activities of different morphogen systems. Subdivision of the ventral neural tube is mediated by the regulation of a set of TFs in response to graded Shh signaling (Figure 3(a)). The boundaries between the expression domains of these TFs are determined by a combination of Shh signaling and cross‐repression between the TFs themselves. Besides inducing expression of these TFs, Shh signaling also feeds back on itself by inducing expression of its receptor Ptch1 and Hedgehog‐interacting protein (Hhip1), two pathway antagonists that bind and sequester Shh protein^87^, ^88^, ^89^, ^90^, ^91^ (Figure 3(c)). This restricts Shh movement and inhibits Shh signaling. Furthermore, Shh signaling desensitizes receiving cells by downregulation of Gas1, Boc, and Cdo, which act as Shh coreceptors.^92^, ^93^, ^94^, ^95^ An outcome of this negative feedback is that cells close to the source, which received high levels of Shh signaling, express high levels of pathway antagonists. In contrast cells further away from the source express high levels of Shh coreceptors, and are sensitized to the signal. In this way the Shh morphogen gradient over time generates an oppositely shaped sensitivity gradient in receiving cells, which uncouples ligand concentration from signaling output of the pathway.^57^, ^84^ Furthermore, Shh pathway adaptation transforms higher levels of ligand into longer duration of signaling (Figure 3(b)). Initially, cells throughout the neural tube are sensitive to Shh and relatively low Shh concentrations are sufficient to strongly activate the pathway. With time negative feedback, through the induction of Ptch1 and Hhip1, desensitizes cells to the signal (Figure 3(c)). Thus, continuously increasing concentrations of Shh are required to overcome inhibition of negative pathway regulators and maintain intracellular pathway activation. In the Shh gradient cells close to the source are exposed to higher levels of Shh ligand, and consequently Shh signaling remains active for longer in these cells (Figure 3(b)). Due to the architecture of the Shh controlled GRN, cells closer to the source not only require higher levels but also longer durations of Shh input.^84^, ^96^ Thus, this mechanism essentially allows cells to integrate both levels and duration of Shh signaling to control differential gene expression.

#### *Pathway Adaptation of the TGFβ Pathway Provides a Way to Measure the Rate of Ligand Increase*

In the case of TGFβ, signaling leads to receptor mediated phosphorylation of Smad2/3 (R‐Smads) that then form a complex with Smad4, translocate to the nucleus and activate target genes (Figure 3(d)).^81^ Importantly, phosphorylation of R‐Smads is also necessary for Smad4 to accumulate in the nucleus and their nuclear localization closely reflects the ligand levels cells are exposed to even over extended periods of time. In contrast, the transcriptional response to TGFβ signaling appears to display temporal adaptation.^85^, ^86^ What is the mechanistic basis of this adaptive behavior? Warmflash et al.^86^ found that Smad4 nuclear translocation in response to TGFβ signaling is only transient and coincides with the transcriptional response (Figure 3(d)). The duration of these bursts of nuclear Smad4 localization is stereotypic (~4 h in mammalian cell culture models and *Xenopus* embryos) and does not reflect levels or duration of pathway stimulation. Instead cytoplasmic relocalization depends on *de novo* protein synthesis, but not ubiquitin‐mediated protein degradation. The molecular details of this mechanism remain to be determined and it seems unlikely that this is the only mechanism at work, as mice expressing a constitutively nuclear version of Smad4 are viable.^97^

Sorre et al.^85^ propose that adaptation of the TGFβ pathway is used to measure the rate with which a ligand increases. If the rate of ligand increase is slow, negative feedback dampens pathway activity and thus the output of the signaling pathway will be low (Figure 3(e)). In contrast, a sudden increase in ligand concentration will result in high levels of signaling (Figure 3(e)). Consistent with this model, the nuclear to cytoplasmic ratio of Smad4 is strongly increased when TGFβ concentrations are abruptly changed compared to a more gradual increase.^85^ Furthermore pathway adaptation can be used to discern pulsatile and constant ligand exposure and thereby allow reuse of the signal. If ligand exposure is persistent, the pathway will stay in the adapted state, while pulsatile ligand exposure with a slower frequency than the rate of adaptation will release the pathway from adaptation and permit reuse of the signal.^85^

### Signal Interpretation via Gene‐Regulatory Networks

#### *GRNs Sharpen the Morphogen Response and Result in Stable Expression Domains*

Morphogen signaling results in the differential activation of target genes. In the canonical view the induction of target genes relies on differential binding affinities of the transcriptional effector for CRMs of the target genes.^98^ In this view, short‐range targets are controlled by CRMs with lower binding affinities than those activated at longer range. It is clear that this is an oversimplified view of morphogen gene regulation. Notably, several studies have failed to find a good correlation between binding affinities of CRMs and distance of target gene induction.^26^, ^28^, ^99^ Moreover, several morphogen pathways (e.g. Wnt and Hh signaling) use bifunctional transcriptional effectors, which act as both repressors and activators, depending on the activity of the signaling, and bind to the same target CRMs.

How can differential induction of target genes be explained, if differential‐binding affinities of transcriptional mediators for CRMs are not sufficient? Significant progress has been made in understanding the dynamic properties of simple transcriptional circuits,^100^ and categorizing recurring GRN motifs in development.^101^ Systematic *in silico* exploration of simple three‐node transcriptional networks that are capable of generating a morphogen‐like spatial pattern^102^, ^103^ highlighted the potential of GRN interactions, especially incoherent feed‐forward loops,^104^ for morphogen interpretation. Consistent with this, it has been demonstrated in several systems that interactions between the morphogen target genes can account for the spatial and temporal patterns of target gene induction.^84^, ^96^, ^105^, ^106^ Interpretation of graded inputs by GRNs offers several advantages. Transcriptional mechanisms can sharpen the response to a graded signal, thereby providing a means to convert a continuous gradient into all‐or‐none changes in gene expression.^84^, ^105^ Furthermore, morphogen‐mediated patterning often occurs in growing tissues, which quickly exceed the size that can be stably patterned by a graded signal.^107^ Morphogen interpretation by GRNs usually results in the establishment of stable expression domains, which can become independent of the initial signal. Thus GRNs can convert a continuous graded signal into discrete states of target gene expression, which can relay information from the early morphogen gradient to later stages of development.^108^

#### *Feed‐Forward Loops*

**Figure 4:**
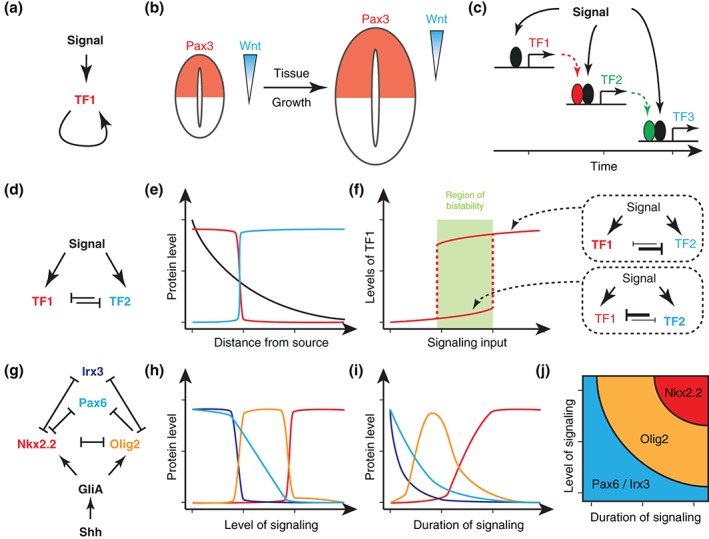
Interpretation of signaling dynamics by gene regulatory networks. (a) Autoregulatory feed‐forward loop. (b) Autoregulatory feed‐forward loops can maintain gene expression domains in growing tissues. In the neural tube, dorsal Pax3 expression is initially induced in response to Wnt signaling, but maintains its own expression as the tissue grows and exceeds the size that can be stably patterned by a morphogen gradient.^109^ (c) Measurement of signal duration by a cascade of feed‐forward loops. Induction of target genes at every step depends on target genes induced previously by the same signal. Thus, target genes induced late in the cascade depend on longer duration of signaling. (d) Mutual cross‐repression of two TFs induced by the same signal. (e) Mutual cross‐repression usually results in the formation of sharp boundaries between gene expression domains. Thus, it provides a way to convert graded input into binary expression of target genes. (f) Mutual cross‐repression usually results in bistability and hysteresis (memory of the signal). Initially, high levels of signaling input are required to induce TF1 as repression of TF1 by TF2 must be overcome (lower line). However, once TF1 is induced much lower levels of signaling are required to maintain its expression as TF2 is repressed by TF1. Thus, there is a region of bistability (green) in which the same level of input favors expression of either TF1 or TF2 depending on the initial conditions. Such a mechanism is useful for maintaining gene expression domains induced in response to adapting signaling pathways or in growing tissues. (g) Gene regulatory network controlling the subdivision of the ventral neural tube into p2, pMN, and p3 progenitor domains in response to Shh signaling (see also Figure 3(a)). (h) Gene expression profile in response to increasing levels of Shh signaling. Upon small increase of Shh signaling, the transcriptional circuit favors expression of Olig2 (orange). In contrast, high levels of Shh signaling lead to expression of Nkx2.2 (red). (i) Temporal dynamics of gene expression for Shh levels favoring Nkx2.2 expression in (h). Although the transcriptional circuit favors Nkx2.2 expression at steady state, it moves through a transient phase with high levels of Olig2 expression. (j) Phase portrait illustrating the connection between levels and duration of Shh signaling for the induction of Olig2 and Nkx2.2 in the ventral neural tube. Induction of Nkx2.2 does not only depend on high levels of Shh pathway activity, but also long duration of signaling.

In its simplest instance these functions can be performed by autoregulatory feed‐forward loops (Figure 4(a)), which can turn a transient signal into stable domains of gene expression. This mechanism has been shown to underlie maintenance of expression of several genes in various systems. Examples include Pax3 expression in the dorsal neural tube, which is initially induced in response to Wnt signaling, but maintained by separate, activating CRMs that positively regulate its own expression^109^ (Figure 4(b)). Similarly, sustained expression of *krox20* in rhombomeres r3 and r5 and expression of *Hoxb3* and *Hoxb4* caudal to the boundary between rhombomeres r6/r7 relies on positive autoregulation.^110^, ^111^

One way to integrate dynamics of signaling is via feed‐forward loops in which a signal elaborates a cascade of target gene expression^51^ (Figure 4(c)). Such a mechanism has been described for the induction of the Dpp target genes *Race* and *C15* during dorsal–ventral patterning in Drosophila.^112^, ^113^ Expression of both these genes relies on input of activated Mad in response to Dpp signaling and the presence of *zerknüllt* (*zen*), another Dpp target gene. Thus, these genes are linked together in a feed‐forward loop where Dpp must first induce *zen* and then the combination of *zen* and continued Dpp signaling induces expression of *Race* and *C15*.^112^, ^113^ Theoretical analysis suggests that timescales in such transcriptional cascades are mainly determined by the initial rate with which target genes are induced and not by the steady state behavior of the GRN^114^ suggesting a way in which the pace of development can be controlled.

#### *Mutual Cross‐Repression*

Another commonly used GRN motif is mutual cross repression, where a signal induces two TFs, which repress each other (Figure 4(d)). Importantly, such systems usually result in bistability and can display sharply delineated threshold responses, allowing cells to turn a graded input into a binary output^115^, ^116^ (Figure 4(e)). This motif has been proposed to underlie differential expression of Gsc and Bra in response to Activin signaling.^117^ Besides generating a binary output, the bistability induced by the cross‐repression means that once the threshold to induce the higher response gene has been crossed, lower levels of signal are sufficient to maintain it (Figure 4(f)).^116^ This provides a mechanism to stabilize gene expression in response to signal fluctuations and acts as an effective memory. The additional recruitment of chromatin modifiers by transcriptional repressors and the epigenetic changes these introduce can extend the timescales of memory beyond the duration of expression of both repressors.

#### *Complex GRNs*

In several systems more complex GRNs have been described (for review see Refs 1, 101, 118). Although the signals that operate upstream of these GRNs and the TFs they comprise are usually not conserved, many of these GRNs seem to follow the same underlying design principles.^1^ (1) The spatial and temporal inputs provided by morphogen signaling serve as inputs into the GRN and, in conjunction with tissue‐specific TFs, determine the state of the transcriptional network. (2) At the molecular level, this is achieved through modular enhancers that control target gene expression and harbor binding sites for multiple GRN components. The regulatory logic of TF binding events at each of these enhancers determines the pattern of gene expression at any given time point within a cell. (3) Finally, GRN dynamics convert the spatial and temporal inputs provided by morphogen signaling into stable patterns of gene expression, thereby converting an analog input (the information provided by the morphogen gradient) into a digital output (the precise domains of gene expression). Note that although the dynamics of morphogen signaling serve as the main governing input into the system, the GRN dynamics are crucial for the readout of the pattern.

One well‐characterized example is the GRN specifying progenitor identity in the vertebrate ventral neural tube. This network illustrates how both levels and duration of a signal are integrated via the regulatory interactions within the GRN to induce differential gene expression. Here, graded input by Shh is converted into expression domains of homeodomain and bHLH TFs, which combinatorially determine progenitor identity.^1^ Boundaries between these domains are positioned by Shh signaling and cross‐repressive interactions between the TFs (Figure 4(g)). This process is best understood for the boundaries between the three most ventral progenitors (p3, pMN, p2), which will give rise to V3 and V2 interneurons and motor neurons. The p3 domain is established by expression of the homeodomain TF Nkx2.2, the pMN domain by the bHLH Olig2 and low levels of Pax6, while p2 progenitors express Irx3 and high levels of Pax6 (Figure 3(a)). These four TFs are linked to each other in a cross‐repressive network, with Nkx2.2 and Olig2 repressing all other TFs, Irx3 repressing Olig2 and Nkx2.2 and Pax6 repressing only Nkx2.2^1^, ^84^, ^96^, ^119^ (Figure 4(g)). Furthermore, Olig2 and Nkx2.2 are activated in response to Shh signaling by Gli proteins (Figure 4(g)) and all members of the network receive basal positive input from uniformly expressed TFs, such as Sox2. Importantly, during development of the neural tube these target genes are activated gradually, with TFs characteristic of more dorsal domains (i.e. Pax6 and Irx3) being expressed first, followed by sequential induction of Olig2 and Nkx2.2.^56^, ^90^, ^120^ Consistent with this, induction of more ventral target genes not only requires higher levels but also longer durations of signaling^7^, ^120^ (Figure 4(h) and (i)). Theoretical and experimental evidence showed that it is the architecture of this transcriptional network in combination with the previously outlined Shh adaptation mechanism that determines both the spatial and temporal response of the target genes.^84^, ^96^ Indeed the way the network responds to Shh imposes an equivalency between the level and duration of Shh signaling for the differential induction of target genes^1^, ^84^, ^119^ (Figure 4(j)).

Taken together, these examples outline how GRN interpretation of morphogens confers several important properties to tissue patterning. (1) GRNs allow the conversion of a graded morphogen input into discrete domains of gene expression. Furthermore cross‐repressive interactions between genes induced in adjacent domains can refine patterning and buffer the patterning system against fluctuations. (2) Positive autoregulation and mutual cross‐repression can confer memory to patterning systems and thereby transform a transient input into a developmentally stable output. (3) Transcriptional feed‐forward cascades and more complex GRNs provide mechanisms for interpreting levels, duration and dynamics of morphogen signaling.

## CONCLUSION

We have outlined in this review how cells diversify their response to morphogens by changing their competence to the signal over time, using the dynamics of signaling and employing transcriptional networks to convert signals into stable patterns of gene expression. Thus to understand development we need to be able to assay with increasing precision the dynamics of signaling and measure the regulation of multiple genes simultaneously. This is necessitating sophisticated experimental approaches. To this end, a wide range of exciting new techniques have emerged that allow the quantification of various aspects of gene regulation in single cells. These range from quantitative imaging approaches to sequencing methods that allow assays of transcriptomes, genome accessibility, and epigenomic modifications. Complementing these, mathematical tools are needed. Current models are largely restricted to relatively simple GRNs. In contrast, the predictive value of models of more complex GRNs is usually limited. Combining theoretical and experimental approaches will likely allow us to construct more detailed and quantitative models and thereby provide a deeper insight into the regulatory logic of the genome.
